# Challenges of optimizing insulin therapy for patients with type 2 diabetes mellitus

**DOI:** 10.1111/jdi.13609

**Published:** 2021-07-19

**Authors:** Fu‐Shun Yen, Chii‐Min Hwu

**Affiliations:** ^1^ Dr. Yen's Clinic Taoyuan Taiwan; ^2^ Department of Medicine Section of Endocrinology and Metabolism Taipei Veterans General Hospital Taipei Taiwan; ^3^ Faculty of Medicine National Yang Ming Chiao Tung University School of Medicine Taipei Taiwan

One hundred years ago, in the early 1920s, Banting and Best discovered insulin. Insulin was first used to treat a dog with its pancreas removed. The dog had a temporary drop in high blood sugar after receiving insulin. The scientists administered insulin to a 14‐year‐old boy with severe diabetes later. His high blood glucose was reduced substantially and urine ketones vanished. The discovery of insulin has set a landmark in the treatment of diabetes mellitus. It is the most effective glucose‐lowering agent, and can quickly control acute and chronic hyperglycemia. Insulin eventually becomes an essential medication for people with diabetes mellitus.

Type 2 diabetes mellitus is a slow progressive disease characterized by a gradual decline in pancreatic β‐cell functions. Many patients with type 2 diabetes require insulin therapy to control the disease. Particularly, Asian patients with type 2 diabetes experience a more rapid decline in β‐cell function and require insulin therapy earlier than their Western counterparts. However, the use of insulin in Asia did not increase to a large degree over the past years, and approximately half of the Asian insulin users did not reach the glycemic target after insulin treatment. Why is insulin therapy suboptimal in patients with type 2 diabetes?

Strategies for implementing effective insulin therapy might cover four crucial steps: (i) initiation; (ii) intensification; (iii) adherence; and (iv) persistence. Initiation is the first stage of insulin therapy. It is common practice to initiate insulin for patients who present with severe hyperglycemia, catabolic features or difficulty maintaining glycemic control by other agents. Intensification might include an increase in insulin dose, the addition of non‐insulin glucose‐lowering medications and/or the supplementation of short‐acting insulin when blood sugar control is suboptimal. Adherence requires patients taking insulin injection following medical advice. Finally, persistence is the continuation of insulin therapy. Patients with high insulin persistence were associated with better long‐term outcomes and lower risks of all‐cause mortality than those with non‐persistence^1^.

Delay of insulin initiation is a serious issue in Asia. A total of 1,065 patients initiating insulin therapy were recently enrolled from Taiwan, Hong Kong, Thailand, Malaysia and the Philippines. Although >70% of the insulin new users were already taking two or more oral agents at the time of insulin initiation, approximately two‐thirds of them had glycated hemoglobin values >9.0%^2^. When patients have hyperglycemia for a long period, they are difficult to treat to therapeutic targets and might develop a high risk of vascular complications.

Insulin initiation might be delayed for many reasons. Insulin distress, an emotional response of the patient to the suggested use of insulin, is a major barrier to insulin initiation^3^. Patients might have injection phobia, fear of hypoglycemia, hold negative self‐perceptions or have misconceptions of insulin use. In contrast, the hurdles presented by healthcare providers in the initiation of insulin therapy are also complex and often overlap with the patient’s concerns. Lack of time for effective patient–doctor communication is an important barrier that needs to be addressed.

Appropriate conversation provides an opportunity to set a positive context for insulin therapy. Experts suggest that people requiring insulin should be counseled individually^3^. A structured education program focusing on simple topics before starting insulin therapy might increase patient retention. A stepwise approach could be followed for insulin‐specific diabetes education. Successful education on insulin therapy relies on effective doctor–patient communication. Clear written instructions might be helpful, and the teach‐back method could be used to confirm the patients’ understanding. National diabetes associations could regularly hold insulin workshops to demonstrate skills for insulin therapy. Mentors from teaching hospitals could travel domestically to discuss insulin therapy with primary care providers.

Initiation of insulin therapy in time is not the only component for adequate glycemic control. Delays in insulin intensification and insufficient dose titration might also cause poor blood glucose control^3^, ^4^. Therapeutic inertia, the failure to advance effective therapies to prevent serious clinical outcomes, can be better understood by the perspectives of providers, patients and healthcare systems^4^. In clinical practice, clinicians might tend to delay insulin dose titration and/or intensification, because they are concerned that the actions might entail consequences; for example, an increased risk of hypoglycemic episodes. Obstacles from patients and healthcare systems also contribute to therapeutic inertia. The patients might have poor awareness of the disease, already take many drugs, fear medication side‐effects or have psychological resistance to insulin. Resource constraints and administrative difficulties with new drugs are two noticeable indicators of therapeutic inertia associated with health systems.

To overcome the barriers of therapeutic inertia, experts suggest implement monitoring systems to assess the overall quality of diabetes care^4^. Applications of simple titration algorithms and educational self‐management programs are integral steps of starting insulin therapy^3^. Insulin intensification inertia can be further addressed by using better insulin preparations with good efficacy and safety profiles, as well as through patient education and effective communication with the patients^3^. In Taiwan, doctors of some hospitals will receive alerts from the hospital computer systems when a patient with a glycated hemoglobin level >9% comes to the clinic. In Singapore, there is a smart phone application that can assist patients to self‐titrate basal insulin by providing recommended daily doses. Importantly, therapeutic inertia is not just the failure to intensify therapy, but also failure to de‐intensify therapy appropriately. Treatment intensification for elderly patients with multiple comorbidities and polypharmacy could increase the risk of falls and cognitive dysfunction. In light of this, clinicians also need to remain sensible in regard to personalized care plans and understand the outcomes of intensification for individual groups of patients.

In a global real‐world survey, one in seven insulin‐treated patients reported that they had ever discontinued insulin for an average duration of 1–2 months for multiple reasons, including influence on social life, cost of test strips and insulin, and lack of support. Nearly 50% of patients who reported poor adherence to insulin treatment in that survey had glycated hemoglobin >9%^5^. Furthermore, non‐persistence of insulin therapy has discernible effects on long‐term clinical outcomes. Recently, we compared mortality risks between patients with or without insulin persistence of >90%. The results showed that those with high insulin persistence were associated with a lower risk of all‐cause mortality than the groups with average or low insulin persistence (adjusted hazard ratio 0.80, 95% confidence interval 0.79–0.81; Figure 1)^1^. Factors associated with insulin non‐persistence were age <40 years, high Charlson Comorbidity Index score, use of multiple oral hypoglycemic agents and severe hypoglycemia incidents during the follow up. Other key factors for non‐persistence are also mentioned in the literature, including treatment complexity, out‐of‐pocket costs and lack of integrated care. Promoting patient self‐management and empowerment might help to improve adherence and persistence with insulin therapy^3^. Using motivational interviewing techniques when discussing insulin therapy is a practical method for patient retention. Advanced technology is also suggested by experts to reduce non‐adherence and non‐persistence^3^. Telephone calls from pharmacists or automated mobile messages would remind patients to refill the prescriptions. Patients with a history of severe hypoglycemia need to monitor their blood glucose regularly, and they should receive insulin regimens with less risk of hypoglycemia ^3^. However, there is no single intervention consistently effective for improving insulin adherence. Novel approaches to enhance long‐term insulin adherence and persistence are widely welcomed.

**Figure 1:**
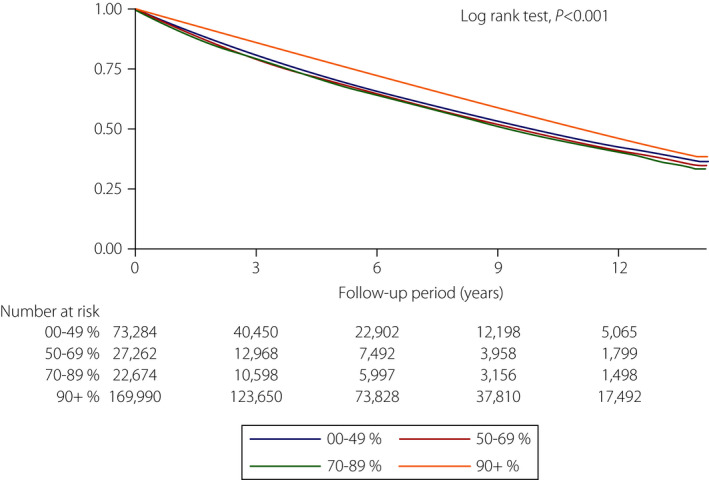
Survival probability estimated by the Kaplan–Meier method among insulin‐treated type 2 diabetes patients with different degrees of insulin persistence (adapted from Figure S1 in Yen *et al*.^1^). A total of 293,210 type 2 diabetes patients who had received insulin therapy were included in a retrospective cohort study based on Taiwan’s National Health Insurance Research Database^1^. In Taiwan, a prescription is usually valid for 90 days. Accordingly, insulin persistence was defined as taking insulin continuously without stopping insulin for >90 days in the study. The degree of insulin persistence was reflected by the portion of time with continual treatment during a 2‐year observation period. Using the propensity score matching technique, mortality rates were compared between patients with or without 90% insulin persistence during the subsequent follow up. Patients with insulin persistence ≥90% were associated with a lower risk of all‐cause mortality than the groups with average or low insulin persistence (log‐rank test, *P* < 0.001).

In summary, although insulin is a potent glucose‐lowering agent, many insulin‐treated patients cannot achieve good glucose control easily. There is a diversity of situations for suboptimal insulin therapy. The disease itself, patient factors and therapeutic inertia are hurdles to be overcome. Although progress has been made, there remains room for improvement of insulin therapy in patients with type 2 diabetes.

## DISCLOSURE

The authors declare no conflict of interest.
